# Biomechanical Evaluation of Suture Augmentation of Dorsal Locking Plate Fixation in Transverse Patella Fractures

**DOI:** 10.5435/JAAOSGlobal-D-24-00194

**Published:** 2024-10-21

**Authors:** Christen E. Chalmers, Min-Shik Chung, Michelle H. McGarry, Thay Q. Lee, John A. Scolaro

**Affiliations:** From the Department of Orthopedic Surgery, University of California Irvine (Dr. Chalmers and Dr. Scolaro); and Orthopaedic Biomechanics Laboratory, Congress Medical Foundation (Mr. Chung, Dr. McGarry, Dr. Lee).

## Abstract

**Introduction:**

Adjunctive suture augmentation of patellar plate fixation has yet to be investigated. Our biomechanical study sought to evaluate whether suture augmentation improves dorsal patellar locking plate fixation. Our hypothesis was that suture augmentation would improve fixation of this construct.

**Methods:**

A transverse patella fracture in six matched cadaveric pairs was stabilized using a patellar locking plate with or without suture augmentation. Specimens were tested at 60° knee flexion with load placed through quadriceps. Cyclic loading followed by load to failure was done. Stiffness, deformation at peak, and nonrecoverable deformation were calculated.

**Results:**

During cyclic loading, suture augmentation demonstrated a higher average stiffness throughout all loads. At the final cycle, deformation was markedly higher without suture augmentation. Average load to failure was higher with suture augmentation. Maximum load to failure occurred at 2500 N in both groups.

**Conclusion:**

Suture augmentation in a transverse patellar fracture model improved dorsal plate fixation, leading to less fracture displacement at the final load. Although suture augmentation demonstrated higher stiffness and lower deformation, these trends were not statistically significant. In both groups, plate fixation sustained very high loads, which reflects the fixation strength of the dorsal locking plate and screw construct in this fracture model.

Patella fractures, although relatively uncommon, can be challenging to treat with a high rate of complications and can result in a high degree of morbidity.^1,2^ Because the patella articulates with the distal femur during knee flexion and plays a critical role in active knee extension, reduction of the articular surface and restoration of the extensor mechanism are imperative for an optimal outcome. For decades, the benchmark of treatment has been the anterior tension band technique, using K-wires or cannulated screws. However, this has been shown to have high complication rates, including a 22% postoperative loss of reduction.^3,4^ A meta-analysis by Zhang et al^5^ compared 949 patients treated with Kirschner wire tension band fixation versus other alternative fixation techniques and overall found no differences in success rate. However, they did find that complications were higher in patients undergoing tension band fixation, whereas alternative fixation techniques led to higher range of motion and functional scores.

Recently, plate fixation of patella fractures has become increasingly common and is an area of interest with promising clinical outcomes.^6,7^ Use of suture material is common during patella fracture fixation because it can decrease implant prominence while helping to resist the deforming muscular forces of the extensor mechanism.^1,8-10^ Innovative plate designs now even include eyelets for suture augmentation, although there is a lack of biomechanical research evaluating the combination of these fixation techniques.

The purpose of this study was to evaluate the effect of tension relieving sutures, placed between a dorsal patellar locking plate and the quadriceps and patellar tendons, on preventing fixation failure in a transverse patella fracture model. We hypothesized that patellar plate fixation that incorporated additional suture fixation into the quadriceps and patellar tendons would be superior to plate fixation alone.

## Methods

**Figure 1:**
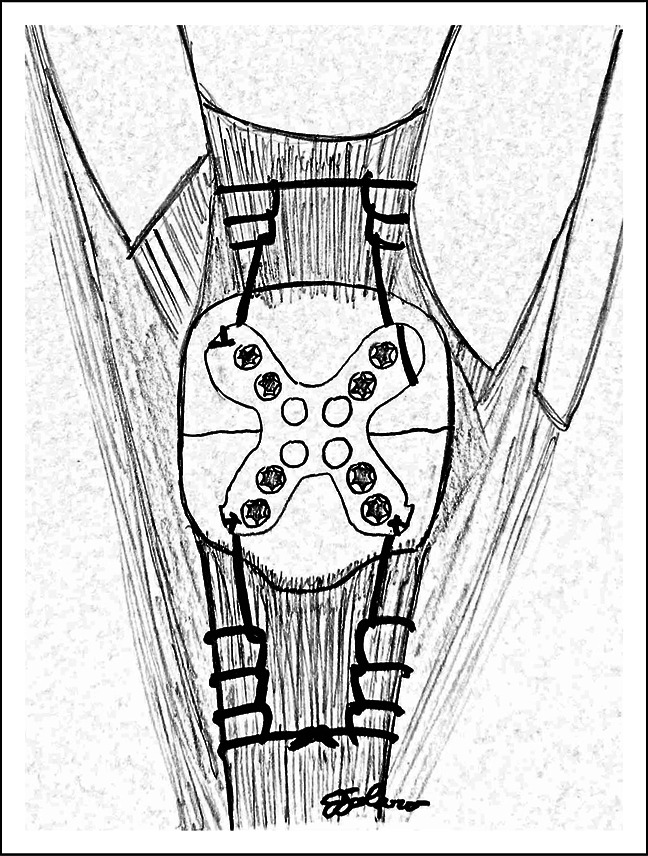
Illustration of suture augmentation of patellar plate fixation of a transverse patella fracture with Krackow locking sutures through quadriceps and patellar tendons.

This biomechanical study was conducted on six matched pairs of fresh frozen cadaveric knees (mean age: 57.0 ± 5.7 years, range: 53 to 64 years). The specimens were dissected to remove all subcutaneous tissue while keeping the collateral ligaments, quadriceps tendon, patella, patellar retinaculum, joint capsule, and patellar tendon intact. The tibial shaft and femoral shaft were potted in polyvinyl chloride pipes with plaster of paris. A transverse patella fracture (AO/OTA 34-C1.1) was created at the measured midpoint of the patella, and the fracture was fixed with a dorsal anatomical patellar locking plate (Endeavor Orthopaedics, Tulsa, OK). One knee of each matched pair underwent dorsal locking plate fixation alone or dorsal locking plate fixation with suture augmentation. Nonabsorbable No. 2 FiberWire sutures were passed from the plate into the quadriceps and patellar tendons using a Krackow locking suture technique (Figure 1). All surgical procedures were performed by a fellowship-trained orthopaedic trauma surgeon. Institutional review board approval was exempt in this biomechanical cadaveric study. Funding for the study was provided through a grant from the Foundation for Orthopaedic Trauma.

**Figure 2:**
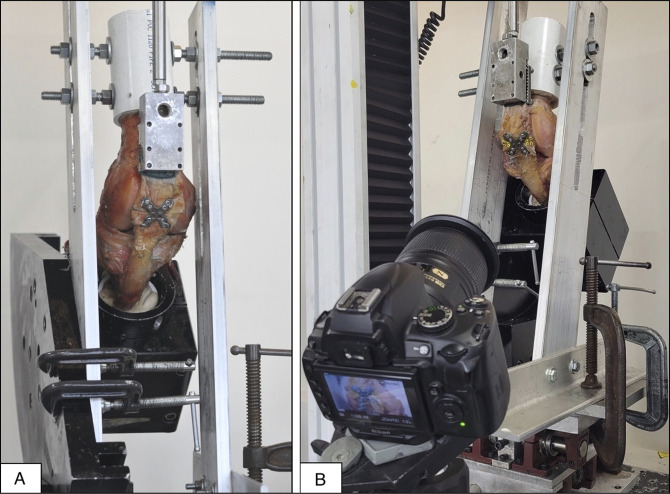
Pictures showing A and B, Testing setup with specimen loaded on Instron machine with video digitizing system (VDS).

The specimens were mounted on an Instron materials testing machine (Instron Corp, Model #3365; Canton, MA) for biomechanical testing. The specimens were mounted with the tibia fixed at 50° flexion and the femur fixed at 10° flexion, for a combined total of 60° knee flexion. On the Instron machine, the tibia was mounted on the x-y translator, whereas the femur was rigidly secured to metal brackets attached to the tibia mount (Figure 2A). Load was applied through the quadriceps tendon by clamping the proximal portion of the quadriceps tendon and attaching this to the loading cell, which was attached to the crosshead of the Instron machine. In all specimens, a custom suture patch was applied at the quadriceps tendon at the interface between the tendon and clamp to prevent pull out of the tendon before fixation failure at high loads. A video digitizing system was used, and each specimen had 4 markers placed with acrylic paint on the medial and lateral aspects of the proximal and distal fracture fragments to allow for video digitizing system analysis of deformation (Figure 2B). Specimens were preloaded with 100 N followed by cyclic loading from 100 to 500 N for 30 cycles at 240 mm/min. Specimens were then loaded for 10 cycles, increasing load by 100 N increments, until failure occurred.

All specimens were able to withstand cyclic loading up to at least 1200 N; therefore, data for cyclic loading are presented between 500 to 1200 N. Stiffness, deformation at peak, and nonrecoverable deformation at each load were measured. Data were averaged for all 12 specimens, and the standard error was calculated. All data are presented as mean ± standard error. Paired t-test was done comparing the locking plate fixation alone with the suture augmentation group using a *P* value of <0.05 to indicate significance.

## Results

**Figure 3:**
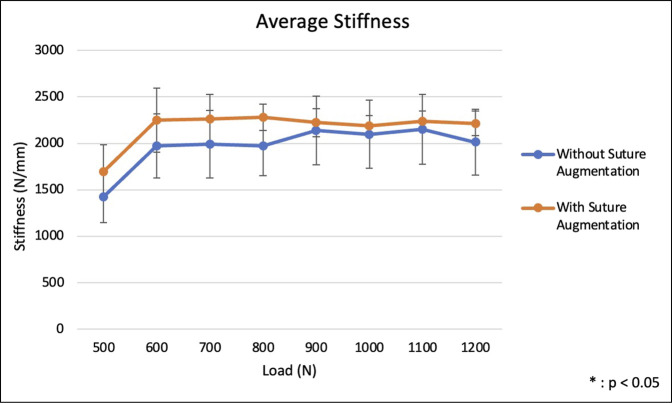
Graph showing average stiffness during cyclic loading at each load from 500 to 1200 N.

**Figure 4:**
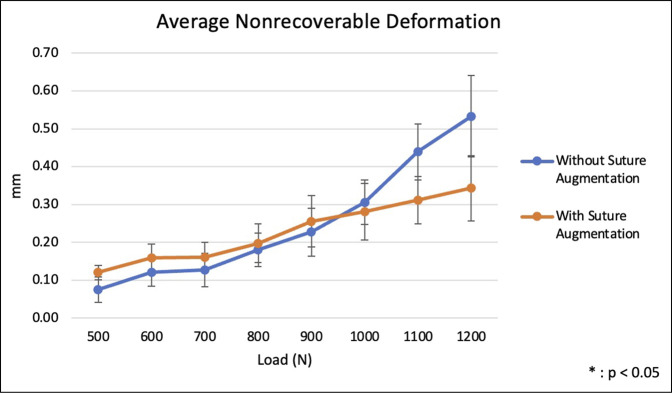
Graph showing average nonrecoverable deformation occurring during cyclic loading from 500 to 1200 N.

**Figure 5:**
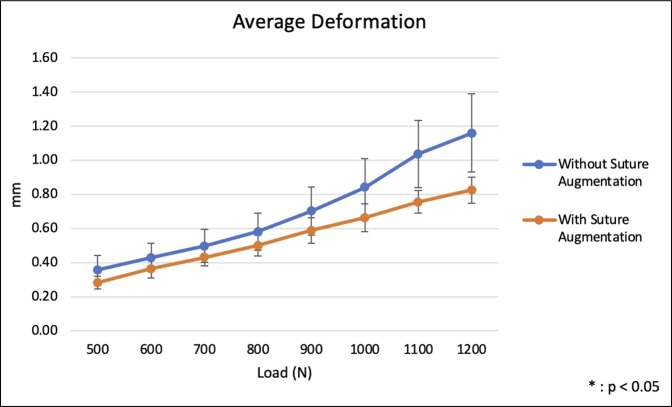
Graph showing average deformation at peak in cyclic loading from 500 to 1200 N.

Throughout cyclic loading from 500 to 1200 N, patellar plate fixation with suture augmentation demonstrated higher average stiffness compared with plate fixation alone. However, no statistically significant differences were observed in stiffness between the groups (*P* > 0.05) (Figure 3 and Table 1). Fixation with suture augmentation showed higher average nonrecoverable deformation at lower loads, whereas the nonaugmented group demonstrated higher average nonrecoverable deformation at higher loads, although these differences did not reach statistical significance (*P* > 0.05) (Figure 4). Average deformation at peak during cyclic loading was not markedly different between the two groups at any load (*P* > 0.05), although the group without suture augmentation did demonstrate larger deformation at all loads (Figure 5).

**Table 1:** Average Stiffness, Deformation at Peak, and Nonrecoverable Deformation at Each Load During Cyclic Loading From 500 to 1200 N With Standard Error

Load (N)	Stiffness (N/mm)		Deformation at Peak (mm)		Nonrecoverable Deformation (mm)	
	Without Suture Augmentation	With Suture Augmentation	Without Suture Augmentation	With Suture Augmentation	Without Suture Augmentation	With Suture Augmentation
500	1422.3 ± 272.5	169.75 ± 284.1	0.4 ± 0.1	0.3 ± 0.0	0.1 ± 0.0	0.1 ± 0.0
600	1971.5 ± 346.8	2251.2 ± 343.9	0.4 ± 0.1	0.4 ± 0.1	0.1 ± 0.0	0.2 ± 0.0
700	1989.8 ± 362.1	2263.6 ± 263.7	0.5 ± 0.1	0.4 ± 0.1	0.1 ± 0.0	0.2 ± 0.0
800	1973.9 ± 319.9	2278.6 ± 142.2	0.6 ± 0.1	0.5 ± 0.1	0.2 ± 0.0	0.2 ± 0.1
900	2140.3 ± 368.5	2222.5 ± 149.1	0.7 ± 0.1	0.6 ± 0.1	0.2 ± 0.1	0.3 ± 0.1
1000	2097.9 ± 368.7	2188.9 ± 108.0	0.8 ± 0.2	0.7 ± 0.1	0.3 ± 0.1	0.3 ± 0.1
1100	2149.4 ± 374.9	2240.2 ± 106.9	1.0 ± 0.2	0.8 ± 0.1	0.4 ± 0.1	0.3 ± 0.1
1200	2012.5 ± 352.9	2215.4 ± 134.3	1.2 ± 0.3	0.8 ± 0.1	0.5 ± 0.1	0.3 ± 0.1

**Figure 6:**
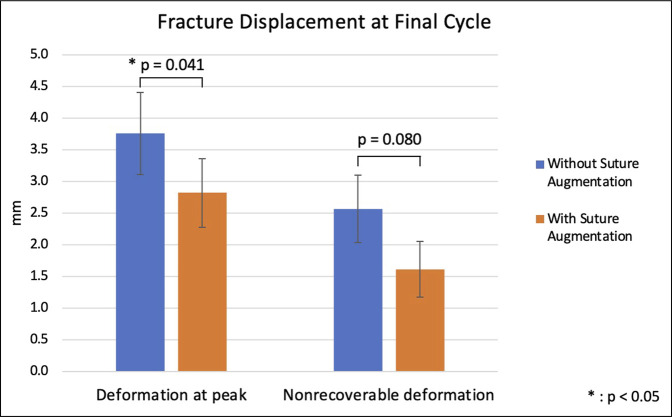
Bar diagram showing average deformation at peak and nonrecoverable deformation at the final cycle before failure.

The highest load to failure occurred at 2500 N for both groups. Average load to failure in the suture augmentation group was 1850.0 ± 183.9 N and was not markedly different from the average load to failure of 1816.7 ± 177.8 N in the group without suture augmentation (*P* = 0.89). The average stiffness in final cycle before failure was 1377.1 ± 97.6 N/mm and 1444.8 ± 280.1 N/mm for fixation with and without suture augmentation, respectively (*P* = 0.80). The average nonrecoverable deformation at peak in final cycle was 1.6 ± 0.4 mm and 2.6 ± 0.5 mm for fixation with and without suture augmentation, respectively (*P* = 0.08). The average deformation at peak in final cycle was 2.8 ± 0.5 and 3.8 ± 0.6 mm for fixation with and without suture augmentation, respectively, which was statistically significant (*P* = 0.04) (Figure 6).

## Surgical Technique

Suture augmentation of patella plate fixation can occur with an implant with preloaded suture (as was done in this biomechanical investigation) or can occur following plate fixation with minifragment implants or a specialized patellar plate, without preloaded suture. The patella fracture is reduced, assessed, and fixed based on the surgeons' preferred technique. The knee is then placed in approximately 45° of flexion to place the extensor mechanism on tension. Following fixation, a #2 (or heavier) nonabsorbable suture is then taken from the plate, or secured to the plate, and passed proximally in into the quadriceps tendon using a locking Krackow suture technique. The suture is then secured back to the plate. Next, a #2 (or heavier) nonabsorbable suture is then taken from the plate, or secured to the plate, and passed distally in into the patellar tendon using a locking Krackow suture technique. The suture is then secured back to the plate. If desired, the distal suture fixation can occur first. The knee is then flexed to 90° to ensure stable fixation before closure. Closure is done according to surgeons' preference. Postoperatively, the patient is allowed full weight-bearing in a knee immobilizer or hinged knee brace locked in extension for 10 to 14 days to allow for wound healing. After the initial wound check, the patient is allowed unrestricted knee range of motion with physiotherapy but maintained in extension for weight-bearing for 6 weeks. At 6 weeks, the brace is removed. Return to unrestricted activity is based on radiographic evaluation and the initial fracture pattern and patient desired level of activity.

## Discussion

Surgical fixation of patella fractures is an area of ongoing research to improve fracture fixation and optimize outcomes. Numerous studies have previously compared fixation methods including tension band constructs, all suture fixation, and various plate fixation techniques with no single method of fixation yielding consistently superior results biomechanically or clinically.^7,11-16^ To date, there has yet to be a biomechanical investigation of the role of suture augmentation in anatomical dorsal locking plate fixation.

In this biomechanical evaluation, suture augmentation in a transverse patellar fracture with dorsal locking plate fixation led to less fracture displacement at the final load. This finding is similar to other studies that have evaluated the use of suture augmentation in fixation of fractures where distracting muscle forces exist.^17^ In our investigation, although suture augmentation demonstrated higher stiffness and lower average deformation than nonaugmented fixation, these trends were not statistically significant and no difference was found in ultimate or average load to failure between the two groups. A potential reason for this was the overall fixation strength of the plate and screw construct, which was able to maintain fixation until very high loads (>1200 N) in both groups. When failure did occur, the suture failed immediately after plate fixation failed, reflecting the fixation strength of the dorsal anatomical locking plate in this transverse fracture model.

Prior biomechanical studies done by Wurm et al and Wagner et al used novel dorsal patellar locking plates with suture eyelets, although both did not incorporate sutures to augment their fixation. Although Wurm et al^7^ found that fixation failure of an anterior patella locking plate occurred at just more than 1000 N in a transverse patella fracture sawbone model, their testing model included an isolated sawbone patella over a plastic pulley. In a cadaveric multifragmentary patellar fracture model, Wagner et al.^14^ found that anterior locking plate fixation demonstrated markedly less fracture displacement following cyclic loading. However, they did not perform load to failure testing and used a maximum force of 200 to 300 N to achieve full extension.

Magister et al. studied the efficacy of incorporating cerclage or Krackow sutures in anterior tension band fixation of a cadaveric transverse fracture model and found that Krackow suture augmentation through the quadriceps and patellar tendons increased the stability of the anterior tension band construct and outperformed the group augmented with cerclage repair.^18^ However, despite Krackow suture augmentation, average load to failure in this construct occurred at 863 N, which is much lower than the 1850 N, the locking plate group with suture augmentation was able to withstand on average in our study.

Our augmented fixation construct highlights the beneficial role that soft-tissue management and neutralization of distracting muscle forces can have in fracture fixation. Because of the deforming forces of the extensor mechanism, despite appropriate fracture fixation, postoperative protocols following patellar fracture fixation often involve immobilization in extension to limit the extensor mechanism for 4 to 6 weeks followed by advancing strength and range of motion as tolerated. The utilization of Krackow sutures affixed to the cranial and caudal portions of the plate, instead of placed around the patella as a cerclage or in retinacular tissue, provides mechanical benefit by resisting the distracting forces of the extensor mechanism that leads to fracture displacement. The placement of the Krackow locking sutures is not technically challenging, is already employed in patellar and quadriceps tendon repair, and does not require additional surgical exposure.

One of the limitations of this study was our transverse fracture model. Although comminuted fractures present more of a clinical challenge, they have been studied less extensively, particularly in biomechanical research. Therefore, we elected to evaluate a transverse fracture model in this initial investigation to allow for comparison of our results to other prior biomechanical investigations. The benefit of suture augmentation into the patellar and quadriceps tendons may be greater in a comminuted patellar fracture model where locking plate fixation alone does not achieve stable fixation. This will be the subject of an upcoming investigation. Another limitation of this study is the time-zero nature that is present in all biomechanical studies.

## Conclusion

In conclusion, this biomechanical study demonstrated that suture augmentation in a transverse patellar fracture model improved dorsal locking plate fixation and led to less fracture displacement at the final load. In addition, both groups fixed with a dorsal locking plate with and without suture augmentation were able to resist markedly high loads without failure, further supporting the use of plate fixation in patella fracture models.
